# A systematic review and Bayesian meta-analysis of the antibiotic treatment courses in AECOPD

**DOI:** 10.3389/fphar.2023.1024807

**Published:** 2023-01-20

**Authors:** Haichuan Yu, Ting Lei, Xiaojie Su, Lu Zhang, Zhouzhou Feng, Xinlong Chen, Jian Liu

**Affiliations:** ^1^ Intensive Care Unit, Lanzhou University First Affiliated Hospital, Lanzhou, China; ^2^ Clinical Medicine Department, First Clinical Medical Academy, Lanzhou University, Lanzhou, China; ^3^ Critical Care Department, Gansu Provincial Maternal and Child Health Hospital, Lanzhou, China

**Keywords:** chronic obstructive pulmonary disease, acute exacerbation, antibiotics, treatment course, Bayesian meta-analysis

## Abstract

**Background:** No consensus exists on the antibiotic treatment course for patients with acute exacerbations of chronic obstructive pulmonary disease (AECOPD). Former studies indicate that shorter courses might have the same efficacy with fewer adverse events, which is inconsistent with guidelines and general practice. Existing evidence allows us to conduct a systematic review and Bayesian analysis on this topic.

**Methods:** Four databases were searched from their inception to January 5, 2023. All statistical estimations were performed using R. “*Gemtc*” was the core package of analysis. CINeMA was used to assess the grade of confidence of the results.

**Results:** Fourteen studies were included in the Bayesian meta-analysis. No difference in the clinical success rate of antibiotic treatment was observed from a super short course (1–3 days) to a long course (≥10 days). Considering the adverse events, the short course (4–6 days) might be the safest. The majority of results were of high or moderate confidence grade.

**Conclusion:** Short course might cause the fewest adverse events. The clinical efficacy of antibiotics might not depend on the course length. Undeniably, more systematic explorations are warranted to investigate the clinical application of a shorter course of antibiotic treatment.

## Introduction

Chronic obstructive pulmonary disease (COPD) is the third largest cause of death on a global scale (GBD Chronic Respiratory Disease Collaborators, 2020). Acute exacerbation (AE) is the leading cause of hospitalization and mortality among the COPD population. Bacterial infection attributes to about 50% of AECOPD (Bafadhel et al., 2011). Therefore, antibiotic treatment is the fundamental element of routine therapy in AECOPD (Anthonisen et al., 1987; Saint et al., 1995; Allegra et al., 2001). However, antimicrobial resistance (AMR) is a huge challenge to global healthcare (Antimicrobial Resistance Collaborators, 2022). By leading to substantial additional death and expense, it may cause enormous burden to patients and the society (Cassini et al., 2019). Consequently, AMR and safety concerns caused by drug overuse in AECOPD have been increasingly discussed and have attracted significant attention (López-Campos et al., 2015).

Till date, the antibiotics treatment course for AECOPD has been poorly discussed and implemented. According to the guidelines, 7–10 days is the suggested treating course in most cases of bacterial infection, which has been applied widely in the treatment of AECOPD. For over 2 decades, more studies have shown that a shorter duration of antibiotics might have the same efficacy. This may draw attention to several guidelines. For instance, the Dutch guideline suggests the 7-day-course, and the global initiative for chronic obstructive pulmonary lung disease (GOLD) report 2022 suggests a 5 to 7-day-course (Geijer et al., 1997; GOLD Reports, 2022). However, the main issue with these guidelines is that these suggestions are usually based on minimal evidence. For example, the advice of “5–7-day course” from GOLD 2022 was based on only one randomized controlled trial (RCT) comparing 5–7 days of antibiotic treatment, which is not convincing (Rg and Cj, 2001; GOLD Reports, 2022). With the increasing evidence, two published systematic reviews highlight comparing shorter and longer antibiotic treatment courses in AECOPD. They suggested that a shorter course surely has similar treating efficacy as a longer one with fewer adverse events (Stolbrink et al., 2018; Llor et al., 2022). However, limitations still exist within these studies. For example, they roughly divided the studies into two groups, limiting their meaning for clinical practice. Considering that shorter treating courses generally means lower cost, higher adherence rate, and lower incidence of AMR and adverse events, it should be given further attention and explored in greater depth.

The Bayesian analysis compares direct and indirect relationships between interventions. This enables us to rank and compare interventions without directly comparing them. Noticeably, some related studies indicate that the treating course could be divided into the following four groups: 1) super short course: 1–3 days; 2) short course: 4–6 days; 3) standard course: 7–9 days; and 4) long course: 10 days or more. Thus, a result of Bayesian analysis depending on such a grouping is more clinically applicable.

This systematic review aimed to comprehensively gather RCT evidence and compare the efficacy and safety of different antibiotic treatment courses in AECOPD using Bayesian analysis. Most importantly, the objective is to provide more detailed and practical evidence for clinical practice within this field.

## Materials and Methods

The current study followed the Preferred Reporting Items for Systematic Reviews and Meta-Analyses (PRISMA) statement and was registered on PROSPERO (CRD42022343479) (Page et al., 2021; Haichuan and Ting, 2022). PRISMA checklist for network meta-analysis is provided in the Supplementary Material Table S1.

### Literature search

We searched PubMed, Embase, Cochrane library, and Web of Science to gather studies published from the establishment of the databases to January 5, 2023. Major terms that were used to build the search strategy included “antibiotics,” “COPD,” and “exacerbation” (The complete search strategy of the four databases has been listed in Supplementary Material Table S2). Only English articles were included during the screening phase.

### Literature screening

Two reviewers (Ting Lei and Xiaojie Su) independently conducted the screening process, and a third reviewer (Haichuan Yu) held discussions in case of disagreements. Inclusion criteria: 1) patients with COPD confirmed by spirometry or physician, 2) patients diagnosed with AE by a physician, 3) antibiotics treatment course should be the focus of the study, and 4) all the included studies were RCTs. Exclusion criteria: 1) patients with comorbid conditions and significant infection in systems other than the respiratory system, 2) the antibiotics of interest have been banned for clinical use, and 3) articles without the success rate or adverse events of antibiotic treatment. The reference lists of included studies were also scrutinized for potential studies wrongly omitted during the previous process.

### Data extraction

Two reviewers (Lu Zhang and Zhouzhou Feng) independently extracted the data through a pre-designed data form. The data form consisted of three parts: the essential characteristics of included studies, the risk of bias in the reports, and targeted outcomes. A third reviewer (Haichuan Yu) combined the two versions of the form into one. Cochrane Risk of Bias (ROB) tool 1.0 was utilized in this study. ROB 1.0 contains seven domains, each with a low, medium, or high rating. We aggregated an overall study evaluation based on assessing these domains.

Herein, the included treating courses were divided into four groups: 1) super short course: 1–3 days; 2) short course: 4–6 days; 3) standard course: 7–9 days; and 4) long course: 10 days or more. Moreover, placebo-controlled trials with clearly defined treating courses were also included.

### Statistical analysis

R (version 4.1.3) was used to undergo the complete analysis, and “*Gemtc*” (version 1.0–1) was the major package utilized. First, a network was built using the function “*mtc.network*,” and then a model was generated using “*mtc.model*.” Finally, the Bayesian analysis was conducted using the “*mtc.run*” function. In function “*mtc.model*,” likelihood/link was set as “binom/log” to calculate the log risk ratio (logRR) with 95% credible interval (95%CrI) depending on the dichotomous data gathered. Models were estimated using JAGS (through the “*rjags*” package). Markov Chain Monte Carlo method computed a fixed effect model based on simulations of 5,000 adaptations and 20,000 iterations within the four chains. A league table was generated to present the relationships among all the treating courses. Function “*exp*” was used to calculate RR from LogRR when generating the league table. Furthermore, a relative effect forest plot was provided to visualize the relative effect of different treating courses than the standard one. A potential scale reduced factor (PSRF) value was calculated to quantify the convergence of the iteration. The node-split method was used to test the consistency assumption (when *p* < 0.05, inconsistency seemed significant). Furthermore, “*mtc.anohe*” was used to conduct the test of homogeneity assumption. I^2^> 50% indicated the significance of the heterogeneity. Sensitivity analysis was performed by comparing the random effect model pooling results with the fixed effect model.

Confidence in Network Meta-Analysis (CINeMA) was used to assess the quality of results of the network meta-analysis (Nikolakopoulou et al., 2020). CINeMA can also be used to perform the network meta-analysis under the “*netmeta*” package in R. Therefore, in addition to obtaining the grading results for the confidence of the analysis results, the results of the network meta-analysis received using CINeMA were also compared with the results from the “*gemtc*” package.

## Results

**FIGURE 1:**
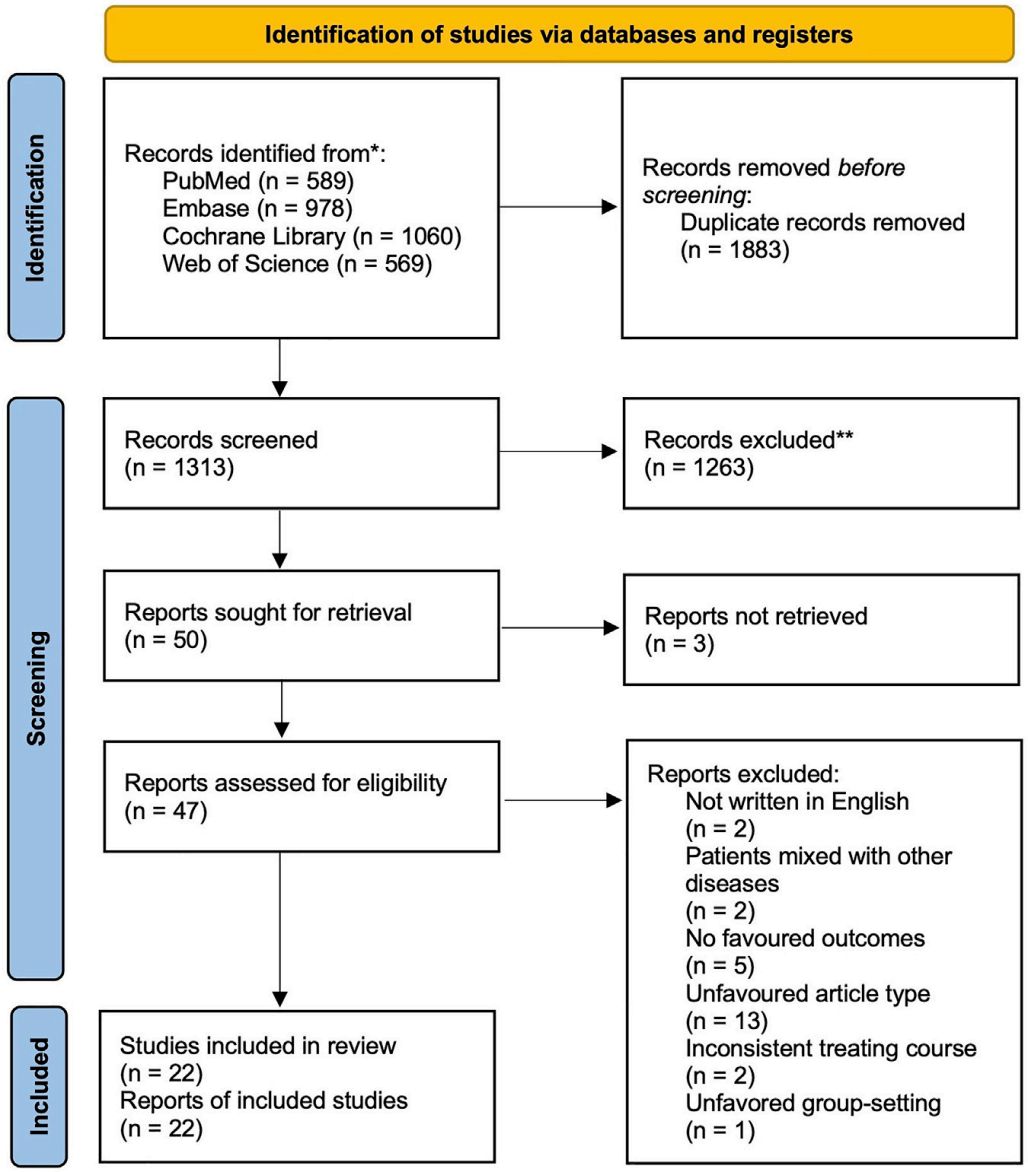
Prisma 2020 flow diagram.

After the search, 3196 articles were included in the screening (search strategy and results are listed in the Supplementary Material Table S2). After discarding the duplicates and excluding the studies that did not match PICOS based on their title and abstract, 50 full articles were retrieved. The full articles were carefully read, and finally, 22 studies with a population of 7934 were included in the Bayesian analysis (essential characteristics of the included studies are presented in Table 1; the PRISMA flow diagram is shown in Figure 1). Most patients were outpatients of similar age. All the articles reported the clinical success rate and adverse effect incidence. 10 studies focused on “chronic bronchitis”, however included patients with airflow limitation. Considering their design would have predated the global use of the term “COPD” so that the population they focused might be consistent with ours, we still included these studies.

**TABLE 1:** Characteristics of included studies.

Study	Region	Patients group	No.	Gender	Comparisons	Age (mean, SD)
Anthonisen et al. (1987)	Canada	Outpatients	116	♀: 23	Trimethoprim-sulfamethoxazole, 160mg/80mg, q12 h Amoxicillin 250mg, q8h or Doxycycline, 100mg, q24h, 10 days	67.3, 9.0
				♂: 93	Placebo, 10 days	
Bennett et al. (1988)	United Kingdom	Inpatients	41	♀: 1	Amoxicillin, 3000 mg, q12h, 3 days	69.3, 1.6
				♂: 40	Amoxicillin, 500 mg, q8h, 7 days	72.4, 1.7
Brusse-Keizer et al. (2014)	Netherlands	Outpatients	35	♀: 14	Co-amoxiclav, 500/125, q8h, 7 days	66.74, 5.49
				♂: 21	Placebo, 7 days	63.87, 8.07
Chodosh et al. (2000)	United States	Outpatients	614	♀: 285	Moxifloxacin, 400 mg, q24h, 5 days	56.9, 15.4
				♂: 329	Moxifloxacin, 400 mg, q24h, 10 days	56.2, 15.8
Daniels et al. (2010)	Netherlands	Inpatients	223	♀: 90	Doxycycline, 7 days	71.0, 10.2
				♂: 133	Placebo, 7days	72.8, 9.2
File et al. (2000)	Multicenter	Outpatients	600	♀: 261	Gemifloxacin, 320 mg, q24h, 5 days	64.2, 11.7
				♂: 339	Co-amoxiclav, 500/125 mg, q8h, 7 days	64.0, 12.1
Gotfried et al. (2005)	Multicenter	Outpatients	485	♀: 250	Clarithromycin, 1,000 mg, q24h, 5 days	62.1, 11.7
				♂: 235	Clarithromycin, 500 mg, q12h, 7 days	61.6, 12.0
Hassan et al. (2015)	Egypt	Outpatients	100	♀: 17	Quinolone, 500mg, q12 h or amoxicillin, 500mg, q8h,10days	60.6, 6.8
				♂: 83	Placebo, 10days	63.0, 6.1
Jørgensen et al. (1992)	Denmark	Outpatients	270	♀: 155	Amoxicillin, 750mg, q12h, 7 days	59.7, NR
				♂: 115	Placebo, 7 days	60.4, NR
Langan et al. (1999)	Multicenter	Outpatients	541	♀: 244	Grepafloxacin, 400 mg, q24h, 5 days	56.8, NR
				♂: 297	Grepafloxacin, 400 mg, q24h, 10 days	56.3, NR
Llor et al. (2012)	Spain	Outpatients	318	♀: 67	Co-amoxiclav, 500/125 mg, q8h, 8 days	68.4, 9.9
				♂: 251	Placebo, 8 days	
Lorenz et al. (1998)	Germany	NR	217	♀: 107	Cefixime, 400 mg, q24h, 5 days	56.0, 14.3
				♂:110	Cefixime, 400 mg, q24h, 10 days	54.0, 12.3
Rg and Cj, (2001)	Multicenter	Outpatients	532	♀: 261	Levofloxacin, 500 mg, q24h, 5 days	60.7, 13.9
				♂: 271	Levofloxacin, 500 mg, q24h, 7 days	59.5, 13.1
Messous et al. (2022)	Tunisia	Inpatients	310	♀: 42	Levofloxacin, 500mg, q24h, 2 days	68.2, 10.5
				♂: 268	Levofloxacin, 500mg, q24h, 7 days	67.1, 10.0
Nouira et al. (2001)	Tunisia	Inpatients	93	♀: 9	Ofloxacin, 400 mg, q24h, 10days	66.2, 6.4
				♂: 84	Placebo, 10 days	66.5, 9.8
Rhee et al. (2015)	Multicenter	Outpatients	342	♀: 30	Zabofloxacin, 367 mg, q24h, 5 days	67.8, 7.8
				♂: 312	Moxifloxacin, 400 mg, q24h, 7 days	68.4, 8.0
Roede et al. (2007)	Netherlands	NR	46	♀: 20	Co-amoxiclav 625 mg, q6h, 3 days	67.9, 13.5
				♂: 26	Co-amoxiclav 625 mg, q6h, 10 days	66.0, 7.9
Sachs et al.(1995)	Netherlands	Outpatients	71	♀: 41	Amoxicillin, 500mg, q8h or Co-trimoxazole, 960mg, q12h, 7 days	51.7, 16.3
				♂: 30	Placebo, 7 days	
Sethi et al. (2005)	Multicenter	NR	893	♀: 315	Co-amoxiclav, 2000/125 mg, q12h, 5 days	60.1, 11.3
				♂: 578	Co-amoxiclav, 875/125 mg, q12h, 7 days	60.3, 11.5
van Velzen et al. (2017)	Netherlands	Outpatients	301	♀: 122	Doxycycline, 100 mg, q12h, 7 days	65.8, 9.3
				♂: 179	Placebo, 7 days	66.4, 9.5
Wilson et al. (2004)	Multicenter	Outpatients	730	♀: 234	Moxifloxacin 400 mg, q24h, 5 days	63.8, 9.7
				♂: 496	Amoxicillin 500 mg, q8h, clarithromycin 500 mg, q12 h, or cefuroxime 250 mg, q12h, all 7 days	62.6, 9.9
Wilson et al. (2012)	Multicenter	Outpatients	1,056	♀: 223	Moxifloxacin, 400 mg, q24h, 5 days	69.6, 6.8
				♂: 833	Co-amoxiclav 875/125 mg, q12h, 10 days	69.3, 6.3

Among the included studies, four compared two or more different antibiotic regimens, nine compared different treating courses with placebo, and the other nine compared various treating courses of the same drugs. Notably, the standard course is the most frequently studied treating course (13 trials), followed by short (10 trials), long (7 trials), and super short (3 trials). Eight trials are multicentered, and studies were mostly conducted in developed areas such as Europe or North America.

17 out of 22 (77.27%) trials included more than 100 patients per group. Inclusion in the report was split 40/60 between the low and medium risk of bias based on the Cochrane ROB (1.0) tool. Based on the comparative analysis of study design, outcome measures, patients involved, and inclusion and exclusion criteria, it was observed that a quantitative synthesis of the evidence using network-based meta-analysis was appropriate. The homogeneity and consistency assumptions were statistically confirmed (see the results of analysis of heterogeneity and inconsistency in Supplementary Data S1).

Before undergoing meta-analysis, it could be roughly judged that in all the included studies, no superiority or inferiority existed between the groups. The overall risk of bias was graded “medium to low.” The primary reason that increased the risk was that some of the included studies could not report the methods for avoiding publication bias (see ROB results in Table 2).

**TABLE 2:** Risk of Bias assessment.

Study	D1	D2	D3	D4	D5	D6	D7
Anthonisen et al. (1987)	+	?	+	+	+	?	+
Bennett et al. (1988)	?	+	+	?	+	?	?
Brusse-Keizer et al. (2014)	+	+	+	?	+	?	+
Chodosh et al. (2000)	+	+	?	?	+	?	?
Daniels et al. (2010)	+	+	+	+	+	+	+
File et al. (2000)	?	?	?	?	+	?	?
Gotfried et al. (2005)	?	+	+	+	+	?	+
Hassan et al. (2015)	?	?	?	?	?	?	?
Jørgensen et al. (1992)	?	?	+	?	+	?	?
Langan et al. (1999)	?	+	?	?	+	+	?
Llor et al. (2012)	+	?	+	+	+	+	+
Lorenz et al. (1998)	?	+	?	?	+	?	?
Rg and Cj, (2001)	?	?	?	?	+	?	?
Messous et al. (2022)	+	+	+	+	+	+	+
Nouira et al. (2001)	+	+	+	+	+	+	?
Rhee et al. (2015)	+	+	+	+	+	+	+
Roede et al. (2007)	+	+	+	+	+	?	+
Sachs et al.(1995)	+	+	+	?	+	?	?
Sethi et al. (2005)	?	?	?	?	+	?	?
van Velzen et al. (2017)	+	+	+	+	+	+	+
Wilson et al. (2004)	+	+	+	+	+	?	+
Wilson et al. (2012)	+	+	+	+	+	+	+

**Notes:** D1, randomization sequence (selection bias); D2, Allocation concealment (selection bias); D3, Blinding of participants and personnel (performance bias); D4, Blinding of outcome assessment (detection bias); D5, Incomplete outcome data (attrition bias); D6, Selective reporting (reporting bias); D7, Other bias. +, low risk; ?, unclear risk; −, high risk.

**FIGURE 2:**
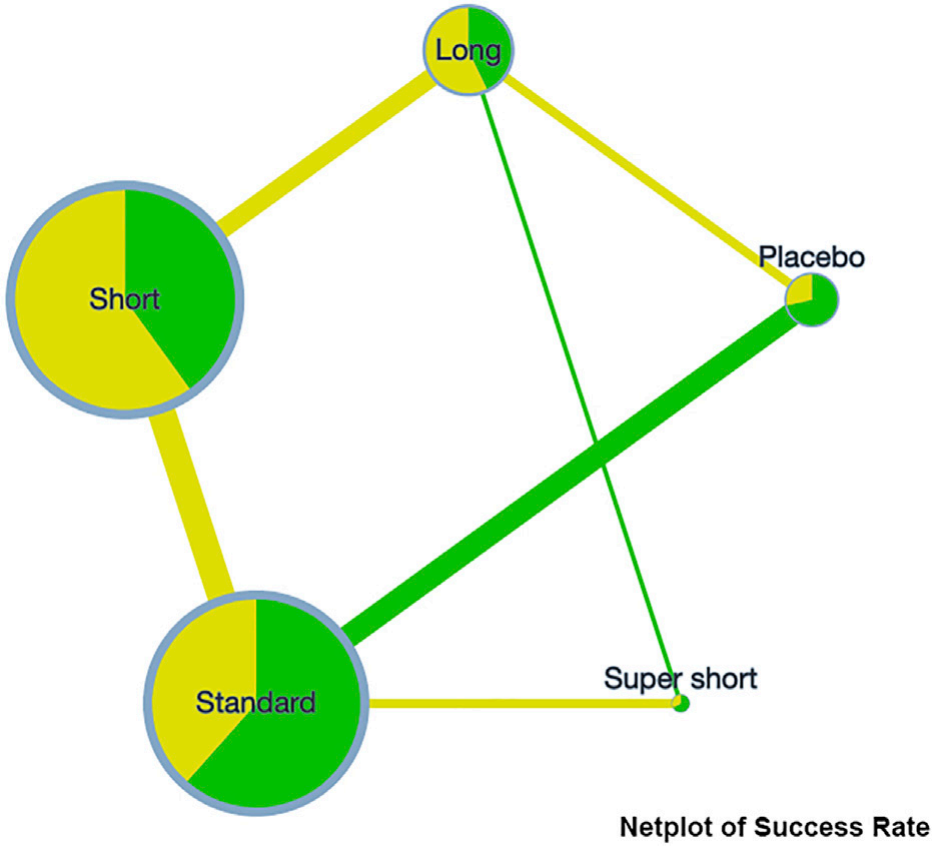
Netplot of success rate.

A network plot was presented on the clinical success rate to show the relationship among the different treatment courses (see Figure 2). Compared to placebo, the RR [95%CrI] of each course are standard, 1.11 [1.04, 1.18], super short, 1.17 [1.03, 1.33]; short, 1.12 [1.05, 1.19]; and long, 1.13 [1.06, 1.22]. This indicates that any course of antibiotics might cause benefit to COPD exacerbation. Detailed comparative analysis has been presented in the league table, and no significant difference could be observed among the different treating courses (see Table 3).

**TABLE 3:** League table from Bayesian meta-analysis.

Clinical success rate (studies comparing mixed antibiotics not omitted.)				
Standard				
0.95 (0.85, 1.06)	**Super short**			
0.99 (0.96, 1.01)	1.04 (0.93, 1.17)	**Short**		
0.98 (0.94, 1.02)	1.03 (0.92, 1.16)	0.99 (0.96, 1.02)	**Long**	
**1.11 (1.04, 1.18)**	**1.17 (1.03, 1.33)**	**1.12 (1.05, 1.19)**	**1.13 (1.06, 1.22)**	**Placebo**
**Clinical Success Rate** (Studies comparing mixed antibiotics omitted.)				
**Standard**				
0.95 (0.85, 1.06)	**Super short**			
0.97 (0.93, 1.01)	1.03 (0.91, 1.16)	**Short**		
**0.94 (0.89, 1)**	0.99 (0.88, 1.12)	0.97 (0.92, 1.01)	**Long**	
**1.1 (1.04, 1.17)**	**1.16 (1.02, 1.32)**	**1.13 (1.05, 1.22)**	**1.17 (1.08, 1.27)**	**Placebo**
**Adverse Events** (Studies comparing mixed antibiotics not omitted.)				
**Standard**				
2.21 (0.98, 6)	**Super short**			
**1.13 (1.01, 1.27)**	0.51 (0.19, 1.16)	**Short**		
1.07 (0.91, 1.27)	0.49 (0.18, 1.1)	0.95 (0.84, 1.07)	**Long**	
1.12 (0.89, 1.41)	0.51 (0.18, 1.18)	0.99 (0.77, 1.27)	1.04 (0.8, 1.36)	**Placebo**
**Adverse Events** (Studies comparing mixed antibiotics omitted.)				
**Standard**				
2.12 (0.9, 5.52)	**Super short**			
**1.16 (1.02, 1.34)**	0.55 (0.21, 1.29)	**Short**		
0.97 (0.76, 1.24)	0.46 (0.17, 1.09)	0.83 (0.67, 1.03)	**Long**	
1.12 (0.89, 1.42)	0.53 (0.2, 1.28)	0.96 (0.74, 1.25)	1.16 (0.85, 1.58)	**Placebo**

**Note:** Data listed are all presented in form of “RR (95% Credible Interval).

The values in each cell of the table indicate the comparative results of the interventions corresponding to the columns and rows, and bolded values indicate that the result is statistically significant.

**FIGURE 3:**
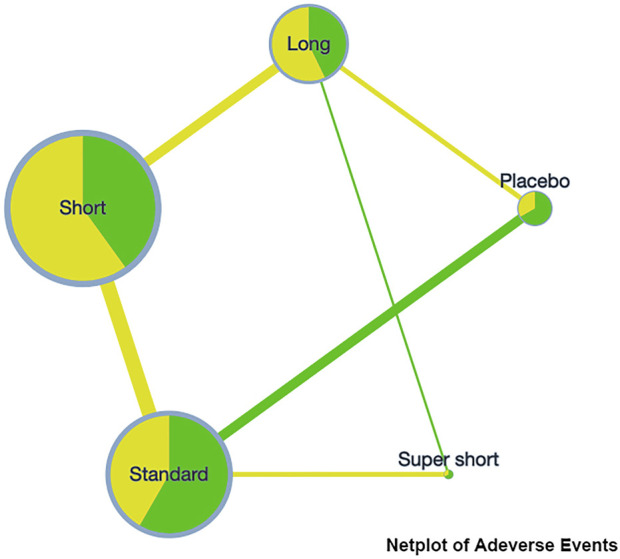
Netplot of adeverse events.

On the incidence rate of adverse events, the network plot was similar to the one with a clinical success rate (see Figure 3). Based on the league table, a short course has a significantly lower risk (RR [95%CrI], 0.88 [0.79, 0.99]) of adverse events compared with the standard course. However, no further difference was observed among the other courses (see Table 3).

Notably, the difference is absent in most cases. Therefore, rank plots and SUCRA were not provided since they could be meaningless under this circumstance.

PSRF value was 1.00 for clinical success rate and 1.01 for adverse events, indicating that the convergence of iteration was satisfying. The node-split analysis results showed no significant inconsistency (all *p* > 0.05). The heterogeneity of the analysis was acceptable (all *I* ^*2*^ < 50%). Results of sensitivity analysis indicated that the results were robust to the different pooling methods (see the results of analysis of heterogeneity and inconsistency in Supplementary Data S1).

Furthermore, the confidence grade of our results was assessed using CINeMA online application. A full assessment was presented in the Supplementary Material S1. All the comparison results were of high confidence. Imprecision was the main reason for downgrading, mainly because the estimated 95%CrI of RR included “1". Reporting this bias raised some concerns since there was no comparison including more than 10 trials, so publication bias could not be tested (Egger et al., 1997). The full assessment is presented in Table 4. Moreover, CINeMA provided a result of the network analysis based on the “*network*” package in R, which was highly similar to what we obtained from the “gemtc” package (see the results of analysis of heterogeneity and inconsistency in Supplementary Data S1).

**TABLE 4:** CINeMA evidence confidence rating results.

	Comparison	Number of studies	Within-study bias	Reporting bias	Indirectness	Imprecision	Heterogeneity	Incoherence	Confidence rating
Clinical success rate	Mixed evidence								
	**Long: Placebo**	2	No concerns	Low risk	No concerns	No concerns	No concerns	No concerns	High
	**Long: Short**	4	Some concerns	Low risk	No concerns	Major concerns	No concerns	No concerns	Low
	**Long: Super short**	1	No concerns	Low risk	No concerns	Major concerns	No concerns	No concerns	Moderate
	**Placebo: Standard**	5	No concerns	Low risk	No concerns	No concerns	No concerns	No concerns	High
	**Short: Standard**	6	Some concerns	Low risk	No concerns	Major concerns	No concerns	No concerns	Low
	**Standard: Super short**	2	No concerns	Low risk	No concerns	Major concerns	No concerns	No concerns	Moderate
	**Indirect evidence**								
	**Long: Standard**	—	Some concerns	Low risk	No concerns	Major concerns	No concerns	No concerns	Low
	**Placebo: Short**	—	No concerns	Low risk	No concerns	No concerns	No concerns	No concerns	High
	**Placebo: Super short**	—	No concerns	Low risk	No concerns	No concerns	No concerns	No concerns	High
	**Short: Super short**	—	No concerns	Low risk	No concerns	Major concerns	No concerns	No concerns	Moderate
**Adverse Events**	**Mixed evidence**								
	**Long: Placebo**	2	Some concerns	Low risk	No concerns	Major concerns	No concerns	No concerns	Low
	**Long: Short**	4	No concerns	Low risk	No concerns	Major concerns	No concerns	No concerns	Moderate
	**Long: Super short**	1	No concerns	Low risk	No concerns	Major concerns	No concerns	No concerns	Moderate
	**Placebo: Standard**	4	No concerns	Low risk	No concerns	Major concerns	No concerns	No concerns	Moderate
	**Short: Standard**	6	Some concerns	Low risk	No concerns	No concerns	Major concerns	No concerns	Low
	**Standard: Super short**	2	No concerns	Low risk	No concerns	Major concerns	No concerns	No concerns	Moderate
	**Indirect evidence**								
	**Long: Standard**	—	Some concerns	Low risk	No concerns	Major concerns	No concerns	No concerns	Low
	**Placebo: Short**	—	No concerns	Low risk	No concerns	Major concerns	No concerns	No concerns	Moderate
	**Placebo: Super short**	—	No concerns	Low risk	No concerns	Major concerns	No concerns	No concerns	Moderate
	**Short: Super short**	—	No concerns	Low risk	No concerns	Major concerns	No concerns	No concerns	Moderate

Furthermore, considering four studies with two or more different antibiotic regimens, the Bayesian analysis was rerun after omitting the studies. No significant difference between the two results was observed (see Table 3).

## Discussion

This systematic review and Bayesian meta-analysis found that it is not “the longer, the better” when treating AECOPD with antibiotics. In particular, super short or short courses were not worse than standard or long courses in clinical success rate. Moreover, a short course (4–6 days) might be the safest choice when considering the incidence of adverse events. These results were based on 22 RCTs rated “medium to low risk” using ROB Tools. Four studies included groups with different antibiotics, but after we omitted them, the results did not change significantly.

Shorter courses of antibiotic treatment in AECOPD are not worse in terms of clinical success rate. In this study, the clinical success rate among different courses did not differ. This phenomenon could be attributed to several reasons. Non-etheless, about half of AECOPD is caused by infection, but the other half is for non-infective reasons (Costelloe et al., 2010). Moreover, there is still a lack of cost-effective, reliable, and instant methods to recognize viral infection in the infected population (GOLD Reports, 2022). These two factors lead to uncertainty and controversy about the need to administrate antibiotics in a large population and might contribute to the uncompetitive course.

A shorter course of antibiotic treatment in AECOPD might cause equal or fewer adverse events. A recent systematic review reported that excess antibiotic treatment is commonly used in the respiratory department, which causes significantly more adverse events (Vaughn et al., 2019). In this study, it was found that a short course (4–6 days) led to significantly fewer adverse events, which is coherent with former systematic reviews.

A shorter course of antibiotic treatment in AECOPD might have some other benefits. On the one hand, it has been well known that “more antibiotics, more resistance”; as a result, shorter courses could be an effective way to slow down the development of antibiotic-resistant bacterial strains (Goossens et al., 2005). On the other hand, minimal use of antibiotics is associated with lower treatment costs. The outcomes might be attributed to decreased individual costs and fewer population-based antibiotic resistance (Zhen et al., 2019).

WHO introduced a classification system called “AWaRe” (A, access; Wa, watch; Re, reserve) to guide the use of 180 antibiotics (Organization, 2019). Of the 22 trials included in this study, 11 used “Access” drugs and the remaining 11 used “Watch” drugs. This widespread use of antibiotic class that are not recommended as first-line in exacerbations not necessarily caused by bacterial infections is also a cause for concern. It is reassuring to note that there exists clinical trial examining the application of antibiotics based on evidence of biomarkers reflecting bacterial infection now (Mohamed Amine et al., 2021).

This study has some advantages. For instance, this is the first study using methods of the systematic review and Bayesian meta-analysis to compare the efficacy and safety of different antibiotic treatment courses. By dividing treatment courses into four groups, our study provides more guiding significance for clinical practice than former systematic reviews.

Non-etheless, there remain some limitations in this study: 1) There are only 22 studies included in the analysis, and no comparison had more than 10 trials. Moreover, no direct comparisons existed between long and standard courses. All these issues impaired the certainty of our estimation. 2) Half of the included reports were graded as medium risk of bias, which could impair the strength of evidence. 3) Because of the different mechanisms of action and bactericidal efficacy, the results of comparing different duration of antibiotic courses of different antibiotics remains debatable. 4) Within the study, publication bias could not be tested for included trials of each comparison of less than 10. 5) Many of the studies included in this study are multicentre studies and those that are not also widely spread across Asia, Europe, Africa, and North America. Coupled with the limited number of original studies included, it may not have been possible to conduct subgroup analyses on different regions, especially for low- and middle-income countries (LMICs) which suffered more from AMR.

Based on the discussions mentioned above, particular suggestions have been made for clinical practice and future study: 1) The results of this study should be cautiously interpreted due to its limitations. 2) Shorter courses should be considered more in clinical practice; short courses (4–6 days) would be optimal based on the existing evidence. 3) Notably, 2 days is the shortest course we observed. Many systematic explorations are required to determine whether it is the shortest effective course, which will be carried out in future studies. 3) Possibly, more flexible antibiotic regimens should be developed and tested, for instance, the instant use of antibiotics, symptom (sputum purulence, fever, etc.) based treatment, biomarker (e.g., C-reactive protein) guided medication, among others. 4) As aforementioned, only 50% of AECOPD is caused by bacterial infection, so caution needs to be exercised when administering antibiotics to this population, and the WHO ‘Access’ classification should probably be considered first when using antibiotics empirically. 5) Although it was found in this study that a short course of antibiotic treatment may have similar efficacy and fewer adverse events to a standard course of treatment, this does not mean “the shorter, the better,” as inadequate treatment duration is also an important cause of AMR. Caution should be exercised when using shorter than conventional antibiotic courses, and population-based follow-up should be done to monitor the occurrence of AMR.

## Conclusion

By systematic review and Bayesian meta-analysis, it was found that in terms of the antibiotic treatment course of AECOPD, super short (1–3 days), short (4–6 days), and long course (≥10 days) could have similar treating efficacy with the standard course (7–9 days). Short course possesses statistically significant superiority in adverse events compared to standard ones. The limitations of this study are the need for further studies and keeping cautious when interpreting our results.

## Data Availability

The datasets presented in this study can be found in online repositories. The names of the repository/repositories and accession number(s) can be found in the article/Supplementary Material.
