# Supplementary material for: A systematic review and Bayesian meta-analysis of the antibiotic treatment courses in AECOPD
Source: Front Pharmacol. 2023 Jan 20;14:1024807. doi: 10.3389/fphar.2023.1024807 (PMC9895851; doi:10.3389/fphar.2023.1024807)
Supplement: Supplementary file 2 [file Table2.docx]

**Search strategy and result in PubMed**

| # | Search strategy | Results |
| --- | --- | --- |
| 6 | (#1 OR #2) AND (#3 OR #4) AND #5 | 589 |
| 5 | random*[Title/Abstract] | 1333385 |
| 4 | Anti-Bacterial Agents[MeSH Terms] | 424887 |
| 3 | "Anti Bacterial Agent"[Title/Abstract] OR "Anti Bacterial Agents"[Title/Abstract] OR "Anti Bacterial Compound"[Title/Abstract] OR "Anti Bacterial Compounds"[Title/Abstract] OR "Anti Mycobacterial Agent"[Title/Abstract] OR "Anti Mycobacterial Agents"[Title/Abstract] OR "Antibacterial Agent"[Title/Abstract] OR "Antibacterial Agents"[Title/Abstract] OR "antibiotic"[Title/Abstract] OR "antibiotic agent"[Title/Abstract] OR "antibiotic combination"[Title/Abstract] OR "antibiotic drug"[Title/Abstract] OR "antibiotic ointment"[Title/Abstract] OR "antibiotic residue"[Title/Abstract] OR "antibiotic spectrum"[Title/Abstract] OR "antibiotics"[Title/Abstract] OR "antibiotics and their derivatives"[Title/Abstract] OR "Antimycobacterial Agent"[Title/Abstract] OR "Antimycobacterial Agents"[Title/Abstract] OR "Bacteriocidal Agent"[Title/Abstract] OR "Bacteriocidal Agents"[Title/Abstract] OR "Bacteriocide"[Title/Abstract] OR "Bacteriocides"[Title/Abstract] OR "combined antibiotic"[Title/Abstract] | 406148 |
| 2 | Pulmonary Disease, Chronic Obstructive[MeSH Terms] | 63790 |
| 1 | "Pulmonary Disease, Chronic Obstructive"[Title/Abstract] OR "chronic obstructive lung disease"[Title/Abstract] OR "Airflow Obstruction, Chronic"[Title/Abstract] OR "Airflow Obstructions, Chronic"[Title/Abstract] OR "Chronic Airflow Obstruction"[Title/Abstract] OR "Chronic Airflow Obstructions"[Title/Abstract] OR "chronic airway obstruction"[Title/Abstract] OR "Chronic Obstructive Airway Disease"[Title/Abstract] OR "Chronic Obstructive Pulmonary Disease"[Title/Abstract] OR "Chronic Obstructive Pulmonary Diseases"[Title/Abstract] OR "chronic obstructive bronchopulmonary disease"[Title/Abstract] OR "chronic obstructive lung disorder"[Title/Abstract] OR "chronic obstructive pulmonary disorder"[Title/Abstract] OR "chronic obstructive respiratory disease"[Title/Abstract] OR "chronic pulmonary obstructive disease"[Title/Abstract] OR "chronic pulmonary obstructive disorder"[Title/Abstract] OR "COAD"[Title/Abstract] OR "COPD"[Title/Abstract] OR "lung chronic obstructive disease"[Title/Abstract] OR "lung disease, chronic obstructive"[Title/Abstract] OR "obstructive chronic lung disease"[Title/Abstract] OR "obstructive chronic pulmonary disease"[Title/Abstract] OR "obstructive lung disease, chronic"[Title/Abstract] OR "pulmonary disorder, chronic obstructive"[Title/Abstract] OR "Chronic bronchitis"[Title/Abstract] OR "emphysema"[Title/Abstract] | 108382 |

**Search strategy and result in Embase**

| # | Search strategy | Results |
| --- | --- | --- |
| 7 | #6 AND 'Article'/it | 978 |
| 6 | (#1 OR #2) AND (#3 OR #4) AND #5 | 1665 |
| 5 | 'random*':ti,ab,kw | 1812636 |
| 4 | 'antibiotic agent'/exp | 1772424 |
| 3 | 'anti bacterial agent':ti,ab,kw OR 'anti bacterial agents':ti,ab,kw OR 'anti bacterial compound':ti,ab,kw OR 'anti bacterial compounds':ti,ab,kw OR 'anti mycobacterial agent':ti,ab,kw OR 'anti mycobacterial agents':ti,ab,kw OR 'antibacterial agent':ti,ab,kw OR 'antibacterial agents':ti,ab,kw OR 'antibiotic':ti,ab,kw OR 'antibiotic agent':ti,ab,kw OR 'antibiotic combination':ti,ab,kw OR 'antibiotic drug':ti,ab,kw OR 'antibiotic ointment':ti,ab,kw OR 'antibiotic residue':ti,ab,kw OR 'antibiotic spectrum':ti,ab,kw OR 'antibiotics':ti,ab,kw OR 'antibiotics and their derivatives':ti,ab,kw OR 'antimycobacterial agent':ti,ab,kw OR 'antimycobacterial agents':ti,ab,kw OR 'bacteriocidal agent':ti,ab,kw OR 'bacteriocidal agents':ti,ab,kw OR 'bacteriocide':ti,ab,kw OR 'bacteriocides':ti,ab,kw OR 'combined antibiotic':ti,ab,kw | 544993 |
| 2 | 'chronic obstructive lung disease'/exp | 160594 |
| 1 | 'pulmonary disease, chronic obstructive':ti,ab,kw OR 'chronic obstructive lung disease':ti,ab,kw OR 'airflow obstruction, chronic':ti,ab,kw OR 'airflow obstructions, chronic':ti,ab,kw OR 'chronic airflow obstruction':ti,ab,kw OR 'chronic airflow obstructions':ti,ab,kw OR 'chronic airway obstruction':ti,ab,kw OR 'chronic obstructive airway disease':ti,ab,kw OR 'chronic obstructive pulmonary disease':ti,ab,kw OR 'chronic obstructive pulmonary diseases':ti,ab,kw OR 'chronic obstructive bronchopulmonary disease':ti,ab,kw OR 'chronic obstructive lung disorder':ti,ab,kw OR 'chronic obstructive pulmonary disorder':ti,ab,kw OR 'chronic obstructive respiratory disease':ti,ab,kw OR 'chronic pulmonary obstructive disease':ti,ab,kw OR 'chronic pulmonary obstructive disorder':ti,ab,kw OR 'coad':ti,ab,kw OR 'copd':ti,ab,kw OR 'lung chronic obstructive disease':ti,ab,kw OR 'lung disease, chronic obstructive':ti,ab,kw OR 'obstructive chronic lung disease':ti,ab,kw OR 'obstructive chronic pulmonary disease':ti,ab,kw OR 'obstructive lung disease, chronic':ti,ab,kw OR 'pulmonary disorder, chronic obstructive':ti,ab,kw OR 'chronic bronchitis':ti,ab,kw OR 'emphysema':ti,ab,kw | 175494 |

**Search strategy and result in Cochrane Library**

| # | Search strategy | Results |
| --- | --- | --- |
| 6 | (#1 OR #2) AND (#3 OR #4) AND #5 | 1060 |
| 5 | 'random*':ti,ab,kw | 1149929 |
| 4 | MeSH descriptor: [Anti-Bacterial Agents] explode all trees | 13046 |
| 3 | ('Anti Bacterial Agent' OR 'Anti Bacterial Agents' OR 'Anti Bacterial Compound' OR 'Anti Bacterial Compounds' OR 'Anti Mycobacterial Agent' OR 'Anti Mycobacterial Agents' OR 'Antibacterial Agent' OR 'Antibacterial Agents' OR 'antibiotic' OR 'antibiotic agent' OR 'antibiotic combination' OR 'antibiotic drug' OR 'antibiotic ointment' OR 'antibiotic residue' OR 'antibiotic spectrum' OR 'antibiotics' OR 'antibiotics and their derivatives' OR 'Antimycobacterial Agent' OR 'Antimycobacterial Agents' OR 'Bacteriocidal Agent' OR 'Bacteriocidal Agents' OR 'Bacteriocide' OR 'Bacteriocides' OR 'combined antibiotic'):ti,ab,kw | 41033 |
| 2 | MeSH descriptor: [Pulmonary Disease, Chronic Obstructive] explode all trees | 6337 |
| 1 | ('Pulmonary Disease, Chronic Obstructive' OR 'chronic obstructive lung disease' OR 'Airflow Obstruction, Chronic' OR 'Airflow Obstructions, Chronic' OR 'Chronic Airflow Obstruction' OR 'Chronic Airflow Obstructions' OR 'chronic airway obstruction' OR 'Chronic Obstructive Airway Disease' OR 'Chronic Obstructive Pulmonary Disease' OR 'Chronic Obstructive Pulmonary Diseases' OR 'chronic obstructive bronchopulmonary disease' OR 'chronic obstructive lung disorder' OR 'chronic obstructive pulmonary disorder' OR 'chronic obstructive respiratory disease' OR 'chronic pulmonary obstructive disease' OR 'chronic pulmonary obstructive disorder' OR 'COAD' OR 'COPD' OR 'lung chronic obstructive disease' OR 'lung disease, chronic obstructive' OR 'obstructive chronic lung disease' OR 'obstructive chronic pulmonary disease' OR 'obstructive lung disease, chronic' OR 'pulmonary disorder, chronic obstructive' OR 'Chronic bronchitis' OR 'emphysema'):ti,ab,kw | 25846 |

**Search strategy and result in Web of Science**

| # | Search strategy | Results |
| --- | --- | --- |
| 4 | #1 AND #2 AND #3 | 569 |
| 3 | (TI=(random*)) OR (AB=(random*)) OR (AK=(random*)) | 2358109 |
| 2 | (TI=((Anti Bacterial Agent) OR (Anti Bacterial Agents) OR (Anti Bacterial Compound) OR (Anti Bacterial Compounds) OR (Anti Mycobacterial Agent) OR (Anti Mycobacterial Agents) OR (Antibacterial Agent) OR (Antibacterial Agents) OR (antibiotic) OR (antibiotic agent) OR (antibiotic combination) OR (antibiotic drug) OR (antibiotic ointment) OR (antibiotic residue) OR (antibiotic spectrum) OR (antibiotics) OR (antibiotics and their derivatives) OR (Antimycobacterial Agent) OR (Antimycobacterial Agents) OR (Bacteriocidal Agent) OR (Bacteriocidal Agents) OR (Bacteriocide) OR (Bacteriocides) OR (combined antibiotic))) OR (AB=((Anti Bacterial Agent) OR (Anti Bacterial Agents) OR (Anti Bacterial Compound) OR (Anti Bacterial Compounds) OR (Anti Mycobacterial Agent) OR (Anti Mycobacterial Agents) OR (Antibacterial Agent) OR (Antibacterial Agents) OR (antibiotic) OR (antibiotic agent) OR (antibiotic combination) OR (antibiotic drug) OR (antibiotic ointment) OR (antibiotic residue) OR (antibiotic spectrum) OR (antibiotics) OR (antibiotics and their derivatives) OR (Antimycobacterial Agent) OR (Antimycobacterial Agents) OR (Bacteriocidal Agent) OR (Bacteriocidal Agents) OR (Bacteriocide) OR (Bacteriocides) OR (combined antibiotic))) OR (AK=((Anti Bacterial Agent) OR (Anti Bacterial Agents) OR (Anti Bacterial Compound) OR (Anti Bacterial Compounds) OR (Anti Mycobacterial Agent) OR (Anti Mycobacterial Agents) OR (Antibacterial Agent) OR (Antibacterial Agents) OR (antibiotic) OR (antibiotic agent) OR (antibiotic combination) OR (antibiotic drug) OR (antibiotic ointment) OR (antibiotic residue) OR (antibiotic spectrum) OR (antibiotics) OR (antibiotics and their derivatives) OR (Antimycobacterial Agent) OR (Antimycobacterial Agents) OR (Bacteriocidal Agent) OR (Bacteriocidal Agents) OR (Bacteriocide) OR (Bacteriocides) OR (combined antibiotic))) | 555804 |
| 1 | (TI=((Pulmonary Disease, Chronic Obstructive) OR (chronic obstructive lung disease) OR (Airflow Obstruction, Chronic) OR (Airflow Obstructions, Chronic) OR (Chronic Airflow Obstruction) OR (Chronic Airflow Obstructions) OR (chronic airway obstruction) OR (Chronic Obstructive Airway Disease) OR (Chronic Obstructive Pulmonary Disease) OR (Chronic Obstructive Pulmonary Diseases) OR (chronic obstructive bronchopulmonary disease) OR (chronic obstructive lung disorder) OR (chronic obstructive pulmonary disorder) OR (chronic obstructive respiratory disease) OR (chronic pulmonary obstructive disease) OR (chronic pulmonary obstructive disorder) OR (COAD) OR (COPD) OR (lung chronic obstructive disease) OR (lung disease, chronic obstructive) OR (obstructive chronic lung disease) OR (obstructive chronic pulmonary disease) OR (obstructive lung disease, chronic) OR (pulmonary disorder, chronic obstructive) OR (Chronic bronchitis) OR (emphysema))) OR (AB=((Pulmonary Disease, Chronic Obstructive) OR (chronic obstructive lung disease) OR (Airflow Obstruction, Chronic) OR (Airflow Obstructions, Chronic) OR (Chronic Airflow Obstruction) OR (Chronic Airflow Obstructions) OR (chronic airway obstruction) OR (Chronic Obstructive Airway Disease) OR (Chronic Obstructive Pulmonary Disease) OR (Chronic Obstructive Pulmonary Diseases) OR (chronic obstructive bronchopulmonary disease) OR (chronic obstructive lung disorder) OR (chronic obstructive pulmonary disorder) OR (chronic obstructive respiratory disease) OR (chronic pulmonary obstructive disease) OR (chronic pulmonary obstructive disorder) OR (COAD) OR (COPD) OR (lung chronic obstructive disease) OR (lung disease, chronic obstructive) OR (obstructive chronic lung disease) OR (obstructive chronic pulmonary disease) OR (obstructive lung disease, chronic) OR (pulmonary disorder, chronic obstructive) OR (Chronic bronchitis) OR (emphysema))) OR (AK=((Pulmonary Disease, Chronic Obstructive) OR (chronic obstructive lung disease) OR (Airflow Obstruction, Chronic) OR (Airflow Obstructions, Chronic) OR (Chronic Airflow Obstruction) OR (Chronic Airflow Obstructions) OR (chronic airway obstruction) OR (Chronic Obstructive Airway Disease) OR (Chronic Obstructive Pulmonary Disease) OR (Chronic Obstructive Pulmonary Diseases) OR (chronic obstructive bronchopulmonary disease) OR (chronic obstructive lung disorder) OR (chronic obstructive pulmonary disorder) OR (chronic obstructive respiratory disease) OR (chronic pulmonary obstructive disease) OR (chronic pulmonary obstructive disorder) OR (COAD) OR (COPD) OR (lung chronic obstructive disease) OR (lung disease, chronic obstructive) OR (obstructive chronic lung disease) OR (obstructive chronic pulmonary disease) OR (obstructive lung disease, chronic) OR (pulmonary disorder, chronic obstructive) OR (Chronic bronchitis) OR (emphysema))) | 158049 |
