# Supplementary material for: A systematic review and Bayesian meta-analysis of the antibiotic treatment courses in AECOPD
Source: Front Pharmacol. 2023 Jan 20;14:1024807. doi: 10.3389/fphar.2023.1024807 (PMC9895851; doi:10.3389/fphar.2023.1024807)
Supplement: Supplementary file 3 [file DataSheet1.docx]

Supplementary Material

*Data S1-Statistical Analysis Protocol and Results*

**A Systematic Review and Bayesian Meta-analysis of the Antibiotic Treatment Courses in AECOPD**

**Haichuan Yu^1,2^, Xiaojie Su^1,2^, Ting Lei^1,2^, Lu Zhang^1,2^, Zhouzhou Feng^1,2^, Hong Guo^1,2^, Jian Liu^1,3,*^.**

^1^Clinical Medicine department, First Medical College, Lanzhou University, Chengguan District, Lanzhou City, Gansu Province, the People's Republic of China.

^2^Critical care department, the first hospital of Lanzhou University, Chengguan District, Lanzhou City, Gansu Province, the People's Republic of China.

^3^Critical care department, Gansu Provincial Maternal and Child Health Hospital, Chengguan District, Lanzhou City, Gansu Province, the People's Republic of China.

*** Correspondence:**Prof. Jian Liu

Phone number: +86 13609354197

Address: 222 Tianshui South Road, Chengguan District, Lanzhou City, Gansu Province, the People's Republic of China.

Email address: medecinliu@sina.com

**I Success Rate**

In this part, we used "gemtc" package(version 1.0-1) in R(version 4.1.3) to conduct the Bayesian Meta analysis.

1. **Building Network**
   1. **Function used:** *mtc.network*
   2. **Results:**


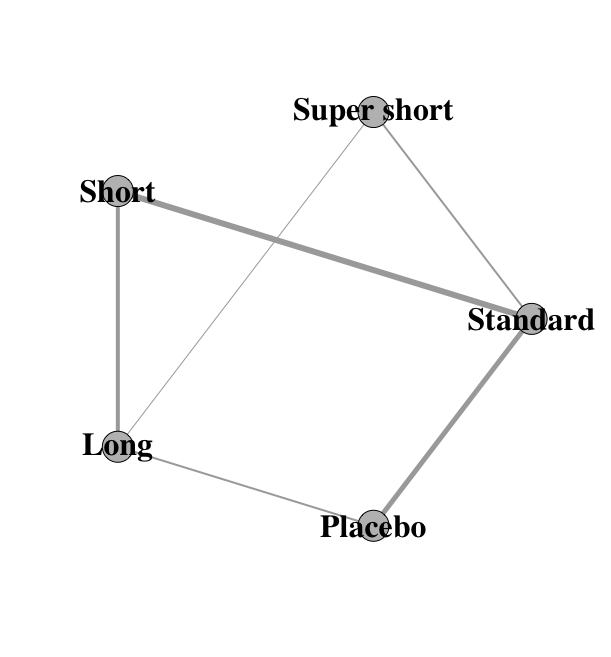


**Figure SI-1 Network of Success Rate**

1. **Modelling and Running**
   1. **Function used:** *mtc.model, mtc.run*
2. **Results Presentation:**
   1. **Forest plot (Placebo was set as the baseline to calculate the relative effect among arms)**


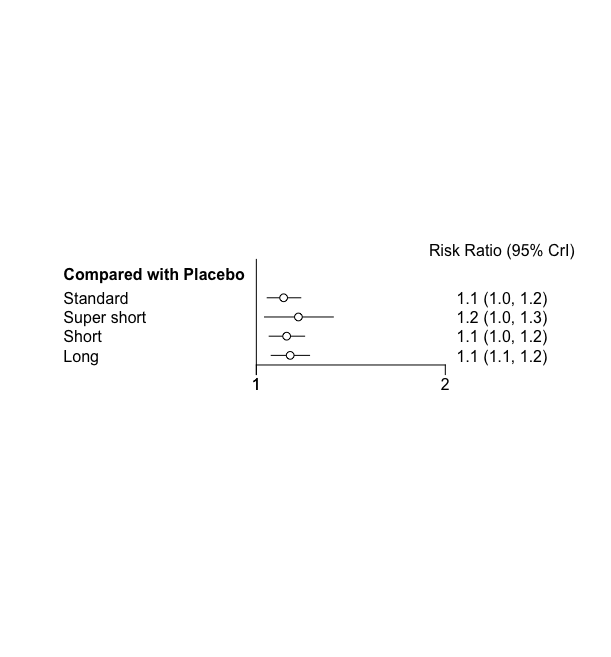


**Figure SI-3.1 Relative Effect Forest Plot of Success Rate (baseline: placebo)**

**Abbreviation:** CrI, credible interval.

- 1. **League table**

**Table SI-3.2 League Table of Success Rate**

| Standard |  |  |  |  |
| --- | --- | --- | --- | --- |
| 0.95 (0.85, 1.06) | Super short |  |  |  |
| 0.99 (0.96, 1.01) | 1.04 (0.93, 1.17) | Short |  |  |
| 0.98 (0.94, 1.02) | 1.03 (0.92, 1.16) | 0.99 (0.96, 1.02) | Long |  |
| **1.11 (1.04, 1.18)** | **1.17 (1.03, 1.33)** | **1.12 (1.05, 1.19)** | **1.13 (1.06, 1.22)** | Placebo |

- 1. **Rank of probability**


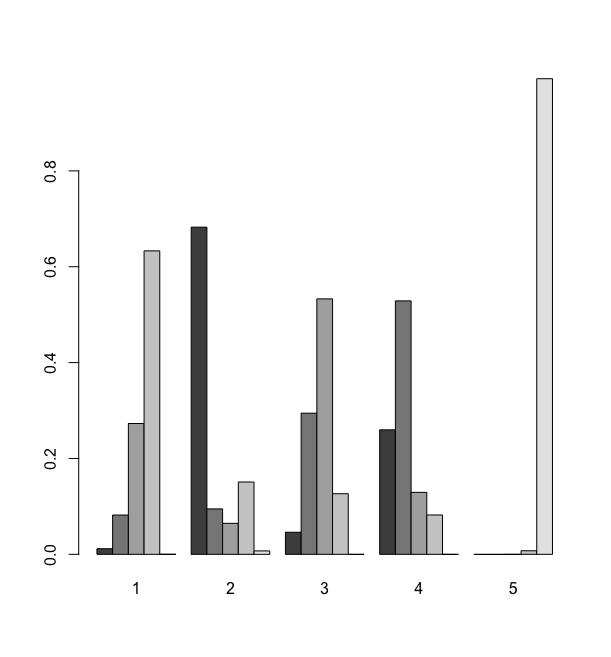


**Figure SI-3.3 Bar chart of rank of probability of success rate.**

**Notes:** 1, standard treating course; 2, super short treating course; 3, short course; 4, long course;5, placebo.

- 1. **SUCRA rank of probability**

**Table SI-3.4 SUCRA rank of probability of success rate.**

| Intervention | SUCRA |
| --- | --- |
| Standard | 36.79% |
| Super short | 82.37% |
| Short | 56.50% |
| Long | 74.14% |
| Placebo | 0.20% |

**Abbreviation:** SUCRA, surface area under the cumulative ranking curve.

1. **Quality Control**
   1. **Convergence test: PSRF value**


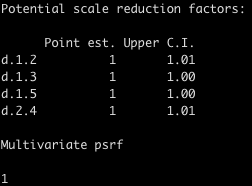


**Figure SI-4.1 PSRF of the Bayesian analysis of success rate**

**Abbreviation:** PSRF, Potential scale reduction factors.

**Note:** The closer the PSRF value is to 1, the better. >1.05 indicates unsatisfactory convergence.

- 1. **Consistency hypothesis: inconsistency test.**
     1. **Function used:** *mtc.nodesplit*
     2. **Results:**

**
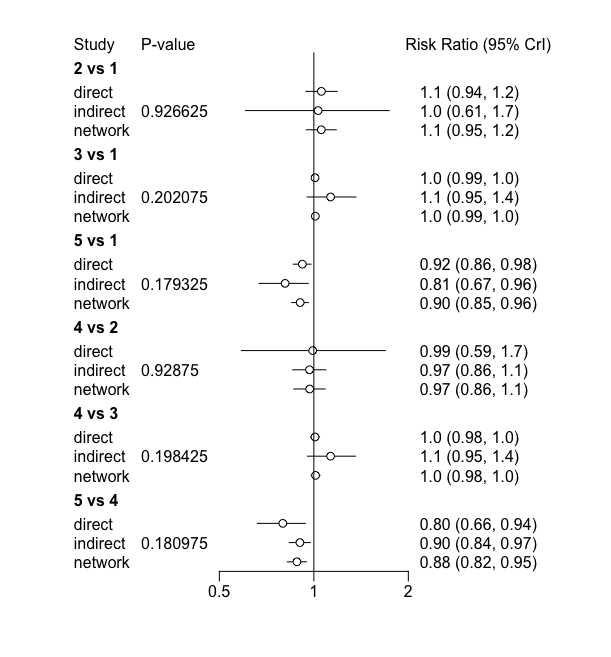
**

**Figure SI-4.2.2 Inconsistency test of the Bayesian analysis of success rate**

**Note:** 1, standard treating course; 2, super short treating course; 3, short course; 4, long course;5, placebo. *P* value > 0.05 indicates that there is no significant inconsistency which means the direct, indirect and network comparison is consistent.

- 1. **Homogeneity hypothesis: Heterogeneity analysis**
     1. **Function used:** *mtc.anohe*
     2. **Results**


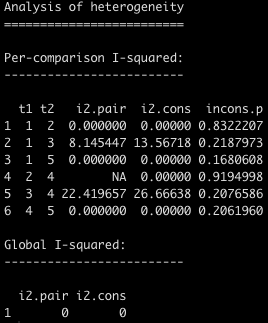


**Figure SI-4.3.2 Heterogeneity analysis of the Bayesian analysis of success rate**

**Note:** From the figure we could see, there exists no significant heterogeneity between any two arms, which allows the use of fixed effect model to estimate the pooling results.

**II Success rate (omitted the trials including mixed interventions)**

1. **Building Network**
   1. **Function used:** *mtc.network*
   2. **Results:**


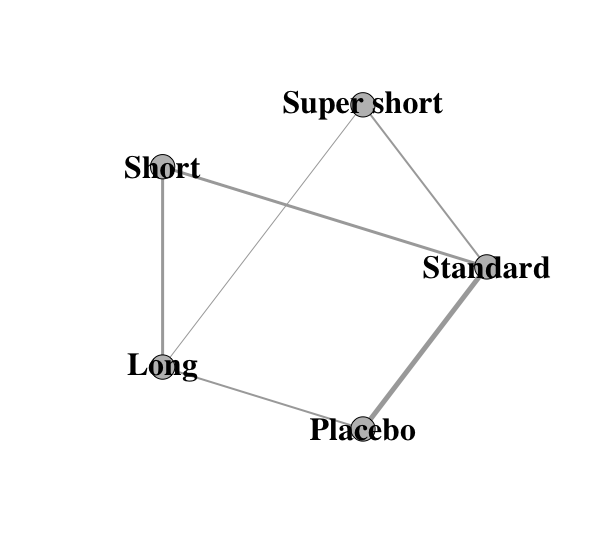


**Figure SII-1 Network of Success rate(omitted the mixed ones)**

1. **Modelling and Running**
   1. **Function used:** *mtc.model, mtc.run*
2. **Results Presentation:**
   1. **Forest plot (Placebo was set as the baseline to calculate the relative effect among arms)**


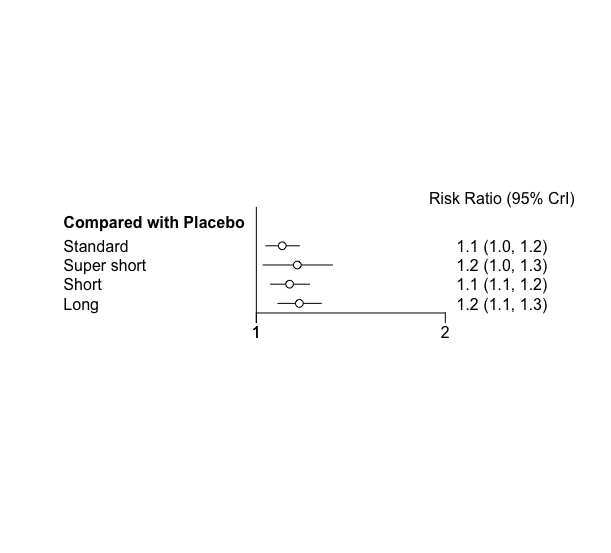


**Figure SII-3.1 Relative Effect Forest Plot of Success rate(omitted the mixed ones) (baseline: placebo)**

**Abbreviation:** CrI, credible interval.

- 1. **League table**

**Table SII-3.2 League Table of Success rate(omitted the mixed ones)**

| Standard |  |  |  |  |
| --- | --- | --- | --- | --- |
| 0.95 (0.85, 1.06) | Super short |  |  |  |
| 0.97 (0.93, 1.01) | 1.03 (0.91, 1.16) | Short |  |  |
| **0.94 (0.89, 1)** | 0.99 (0.88, 1.12) | 0.97 (0.92, 1.01) | Long |  |
| **1.1 (1.04, 1.17)** | **1.16 (1.02, 1.32)** | **1.13 (1.05, 1.22)** | **1.17 (1.08, 1.27)** | Placebo |

- 1. **Rank of probability**


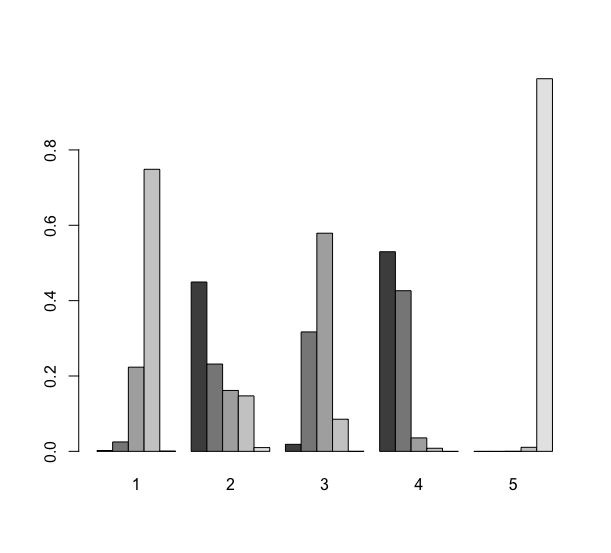


**Figure SII-3.3 Bar chart of rank of probability of success rate(omitted the mixed ones).**

**Notes:** 1, standard treating course; 2, super short treating course; 3, short course; 4, long course;5, placebo.

- 1. **SUCRA rank of probability**

**Table SII-3.4 SUCRA rank of probability of success rate(omitted the mixed ones).**

| Intervention | SUCRA |
| --- | --- |
| Standard | 31.96% |
| Super short | 74.09% |
| Short | 56.71% |
| Long | 86.94% |
| Placebo | 0.29% |

**Abbreviation:** SUCRA, surface area under the cumulative ranking curve.

1. **Quality Control**
   1. **Convergence test: PSRF value**


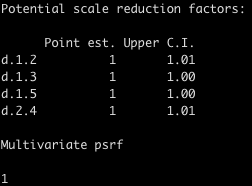


**Figure SII-4.1 PSRF of the Bayesian analysis of success rate(omitted the mixed ones)**

**Abbreviation:** PSRF, Potential scale reduction factors.

**Note:** The closer the PSRF value is to 1, the better. >1.05 indicates unsatisfactory convergence.

- 1. **Consistency hypothesis: inconsistency test.**
     1. **Function used:** *mtc.nodesplit*
     2. **Results:**

**
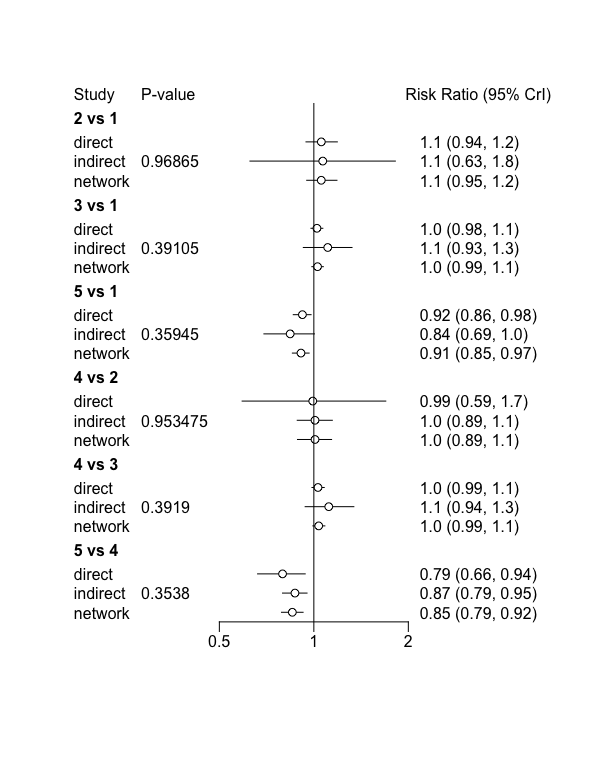
**

**Figure SII-4.2.2 Inconsistency test of the Bayesian analysis of success rate(omitted the mixed ones)**

**Note:** 1, standard treating course; 2, super short treating course; 3, short course; 4, long course;5, placebo. *P* value > 0.05 indicates that there is no significant inconsistency which means the direct, indirect and network comparison is consistent.

- 1. **Homogeneity hypothesis: Heterogeneity analysis**
     1. **Function used:** *mtc.anohe*
     2. **Results**


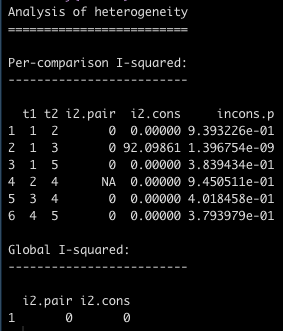


**Figure SII-4.3.2 Heterogeneity analysis of the Bayesian analysis of success rate(omitted the mixed ones)**

**Note:** From the figure we could see, there exists no significant heterogeneity between any two arms, which allows the use of fixed effect model to estimate the pooling results.

1. CINeMA League Table

| **Long** | **1.128 (1.052, 1.208)** | 1.013 (0.981, 1.046) | 1.023 (0.984, 1.064) | 0.969 (0.862, 1.089) |
| --- | --- | --- | --- | --- |
| **0.887 (0.828, 0.950)** | **Placebo** | **0.898 (0.843, 0.957)** | **0.907 (0.854, 0.964)** | **0.859 (0.757, 0.974)** |
| 0.987 (0.956, 1.020) | **1.113 (1.045, 1.186)** | **Short** | 1.010 (0.987, 1.034) | 0.956 (0.854, 1.071) |
| 0.977 (0.940, 1.017) | **1.102 (1.038, 1.171)** | 0.990 (0.967, 1.013) | **Standard** | 0.947 (0.847, 1.058) |
| 1.033 (0.919, 1.161) | **1.164 (1.026, 1.321)** | 1.046 (0.934, 1.171) | 1.056 (0.946, 1.180) | **Super short** |

**II Success rate (omitted the trials including mixed interventions)**

1. **Building Network**
   1. **Function used:** *mtc.network*
   2. **Results:**


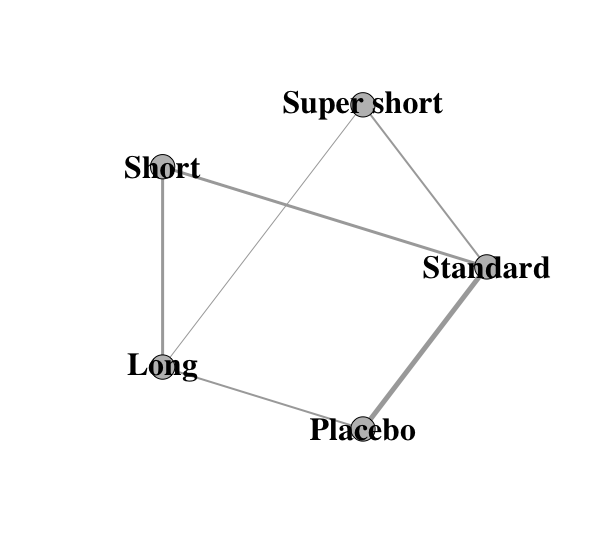


**Figure SII-1 Network of Success rate(omitted the mixed ones)**

1. **Modelling and Running**
   1. **Function used:** *mtc.model, mtc.run*
2. **Results Presentation:**
   1. **Forest plot (Placebo was set as the baseline to calculate the relative effect among arms)**


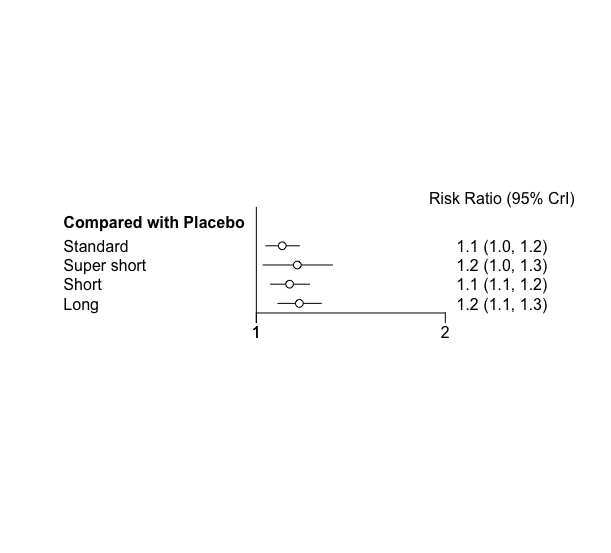


**Figure SII-3.1 Relative Effect Forest Plot of Success rate(omitted the mixed ones) (baseline: placebo)**

**Abbreviation:** CrI, credible interval.

- 1. **League table**

**Table SII-3.2 League Table of Success rate(omitted the mixed ones)**

| Standard |  |  |  |  |
| --- | --- | --- | --- | --- |
| 0.95 (0.85, 1.06) | Super short |  |  |  |
| 0.97 (0.93, 1.01) | 1.03 (0.91, 1.16) | Short |  |  |
| **0.94 (0.89, 1)** | 0.99 (0.88, 1.12) | 0.97 (0.92, 1.01) | Long |  |
| **1.1 (1.04, 1.17)** | **1.16 (1.02, 1.32)** | **1.13 (1.05, 1.22)** | **1.17 (1.08, 1.27)** | Placebo |

- 1. **Rank of probability**


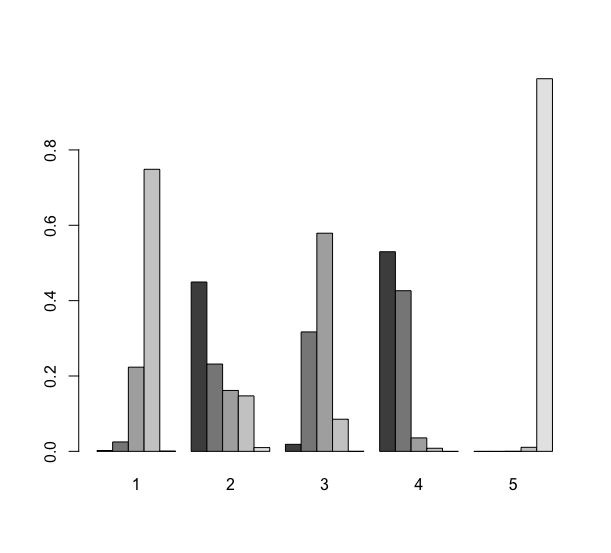


**Figure SII-3.3 Bar chart of rank of probability of success rate(omitted the mixed ones).**

**Notes:** 1, standard treating course; 2, super short treating course; 3, short course; 4, long course;5, placebo.

- 1. **SUCRA rank of probability**

**Table SII-3.4 SUCRA rank of probability of success rate(omitted the mixed ones).**

| Intervention | SUCRA |
| --- | --- |
| Standard | 31.96% |
| Super short | 74.09% |
| Short | 56.71% |
| Long | 86.94% |
| Placebo | 0.29% |

**Abbreviation:** SUCRA, surface area under the cumulative ranking curve.

1. **Quality Control**
   1. **Convergence test: PSRF value**


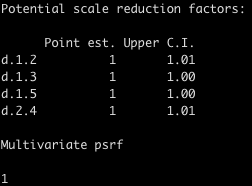


**Figure SII-4.1 PSRF of the Bayesian analysis of success rate(omitted the mixed ones)**

**Abbreviation:** PSRF, Potential scale reduction factors.

**Note:** The closer the PSRF value is to 1, the better. >1.05 indicates unsatisfactory convergence.

- 1. **Consistency hypothesis: inconsistency test.**
     1. **Function used:** *mtc.nodesplit*
     2. **Results:**

**
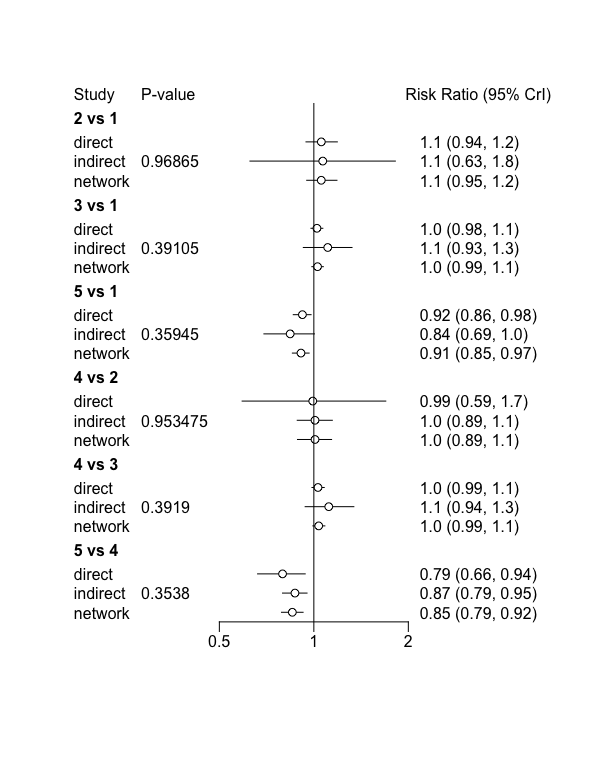
**

**Figure SII-4.2.2 Inconsistency test of the Bayesian analysis of success rate(omitted the mixed ones)**

**Note:** 1, standard treating course; 2, super short treating course; 3, short course; 4, long course;5, placebo. *P* value > 0.05 indicates that there is no significant inconsistency which means the direct, indirect and network comparison is consistent.

- 1. **Homogeneity hypothesis: Heterogeneity analysis**
     1. **Function used:** *mtc.anohe*
     2. **Results**


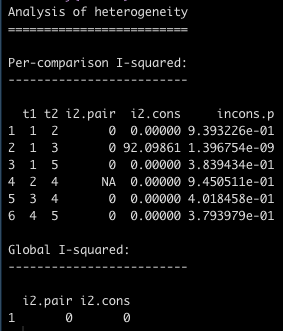


**Figure SII-4.3.2 Heterogeneity analysis of the Bayesian analysis of success rate(omitted the mixed ones)**

**Note:** From the figure we could see, there exists no significant heterogeneity between any two arms, which allows the use of fixed effect model to estimate the pooling results.

**III Adverse events**

In this part, we used "gemtc" package(version 1.0-1) in R(version 4.1.3) to conduct the Bayesian Meta analysis.

1. **Building Network**
   1. **Function used:** *mtc.network*
   2. **Results:**


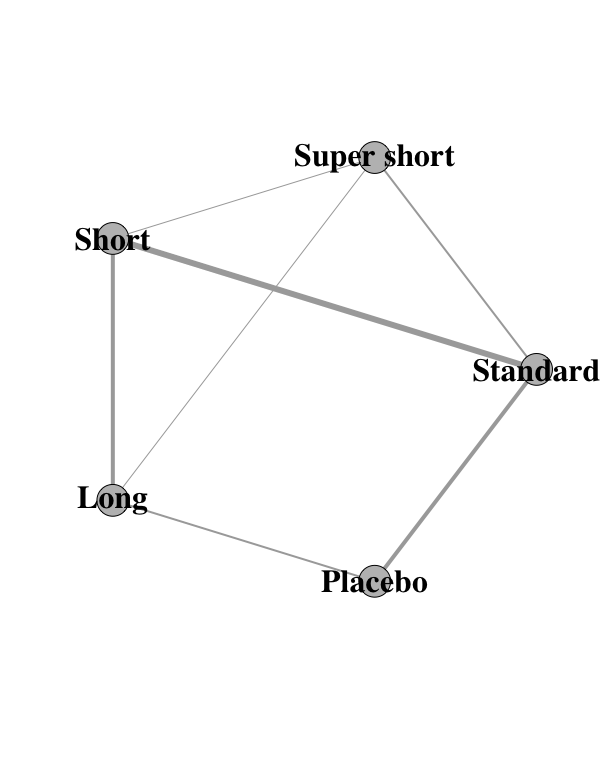


**Figure SIII-1 Network of Adverse events**

1. **Modelling and Running**
   1. **Function used:** *mtc.model, mtc.run*
2. **Results Presentation:**
   1. **Forest plot (Placebo was set as the baseline to calculate the relative effect among arms)**


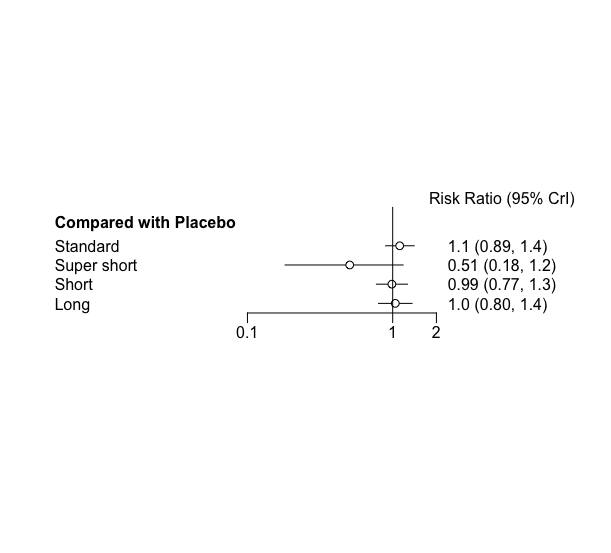


**Figure SIII-3.1 Relative Effect Forest Plot of Adverse events (baseline: placebo)**

**Abbreviation:** CrI, credible interval.

- 1. **League table**

**Table SIII-3.2 League Table of Adverse events**

| Standard |  |  |  |  |
| --- | --- | --- | --- | --- |
| 2.21 (0.98, 6) | Super short |  |  |  |
| **1.13 (1.01, 1.27)** | 0.51 (0.19, 1.16) | Short |  |  |
| 1.07 (0.91, 1.27) | 0.49 (0.18, 1.1) | 0.95 (0.84, 1.07) | Long |  |
| 1.12 (0.89, 1.41) | 0.51 (0.18, 1.18) | 0.99 (0.77, 1.27) | 1.04 (0.8, 1.36) | Placebo |

- 1. **Rank of probability**


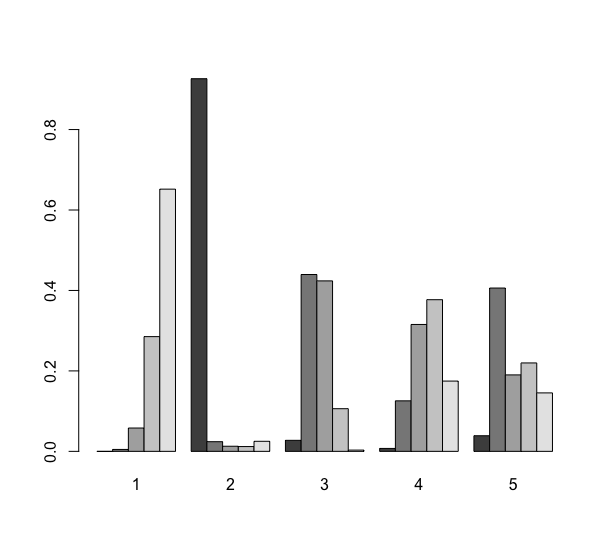


**Figure SIII-3.3 Bar chart of rank of probability of adverse events.**

**Notes:** 1, standard treating course; 2, super short treating course; 3, short course; 4, long course;5, placebo.

- 1. **SUCRA rank of probability**

**Table SIII-3.4 SUCRA rank of probability of adverse events.**

| Intervention | SUCRA |
| --- | --- |
| Standard | 10.40% |
| Super short | 95.37% |
| Short | 59.55% |
| Long | 35.34% |
| Placebo | 49.33% |

**Abbreviation:** SUCRA, surface area under the cumulative ranking curve.

1. **Quality Control**
   1. **Convergence test: PSRF value**


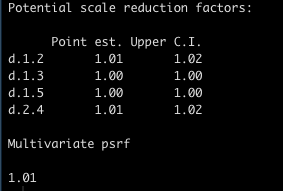


**Figure SIII-4.1 PSRF of the Bayesian analysis of adverse events**

**Abbreviation:** PSRF, Potential scale reduction factors.

**Note:** The closer the PSRF value is to 1, the better. >1.05 indicates unsatisfactory convergence.

- 1. **Consistency hypothesis: inconsistency test.**
     1. **Function used:** *mtc.nodesplit*
     2. **Results:**

**
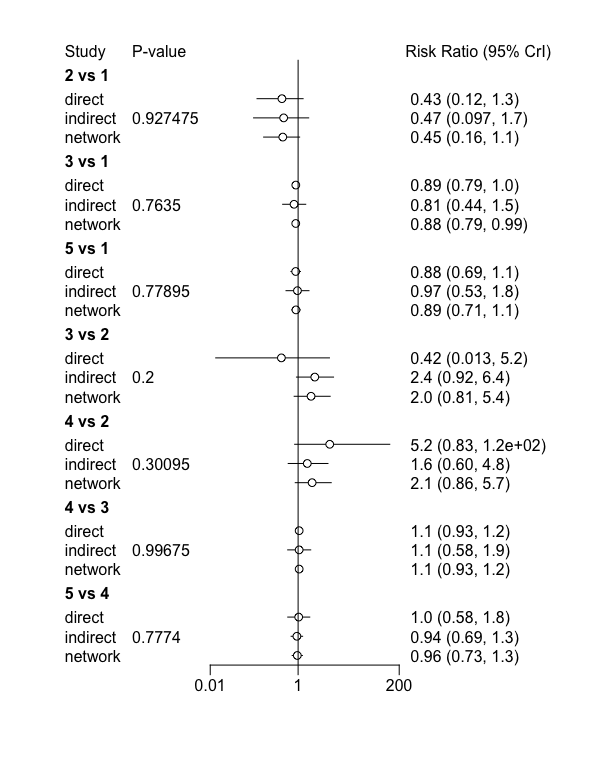
**

**Figure SIII-4.2.2 Inconsistency test of the Bayesian analysis of adverse events**

**Note:** 1, standard treating course; 2, super short treating course; 3, short course; 4, long course;5, placebo. *P* value > 0.05 indicates that there is no significant inconsistency which means the direct, indirect and network comparison is consistent.

- 1. **Homogeneity hypothesis: Heterogeneity analysis**
     1. **Function used:** *mtc.anohe*
     2. **Results**


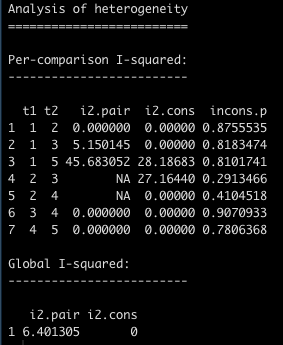


**Figure SIII-4.3.2 Heterogeneity analysis of the Bayesian analysis of adverse events**

**Note:** From the figure we could see, there exists no significant heterogeneity between any two arms, which allows the use of fixed effect model to estimate the pooling results.

1. **CINeMA League table:**

**Table SIII-5 CiNEMA League Table of Adverse Events**

| **Long** | 0.929 (0.641, 1.346) | 0.992 (0.851, 1.156) | 0.914 (0.707, 1.183) | 2.356 (0.742, 7.475) |
| --- | --- | --- | --- | --- |
| 1.076 (0.743, 1.559) | **Placebo** | 1.067 (0.758, 1.503) | 0.984 (0.746, 1.297) | 2.535 (0.782, 8.214) |
| 1.008 (0.865, 1.175) | 0.937 (0.665, 1.320) | **Short** | 0.922 (0.747, 1.138) | 2.375 (0.751, 7.510) |
| 1.094 (0.845, 1.415) | 1.016 (0.771, 1.340) | 1.085 (0.879, 1.338) | **Standard** | 2.576 (0.821, 8.083) |
| 0.424 (0.134, 1.347) | 0.394 (0.122, 1.278) | 0.421 (0.133, 1.331) | 0.388 (0.124, 1.218) | **Super short** |

**IV Adverse events (omitted the trials including mixed interventions)**

1. **Building Network**
   1. **Function used:** *mtc.network*
   2. **Results:**


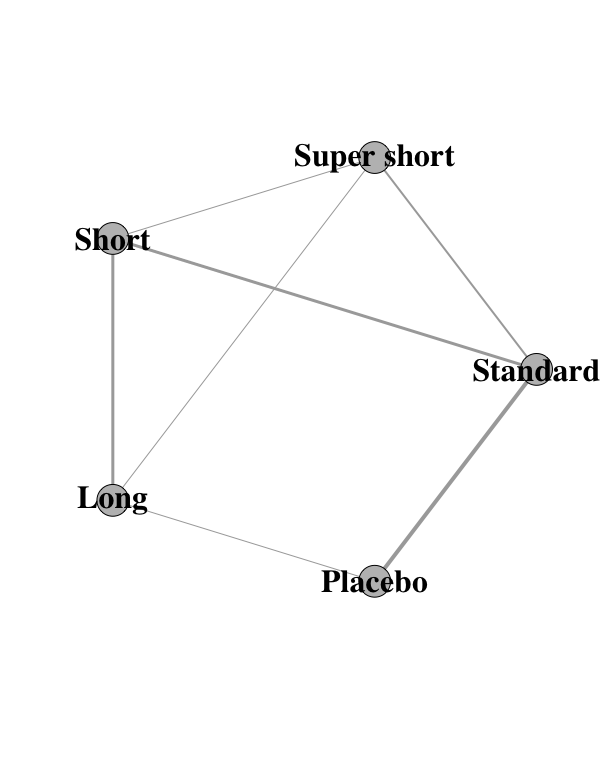


**Figure SIV-1 Network of Adverse events(omitted the mixed ones)**

1. **Modelling and Running**
   1. **Function used:** *mtc.model, mtc.run*
2. **Results Presentation:**
   1. **Forest plot (Placebo was set as the baseline to calculate the relative effect among arms)**


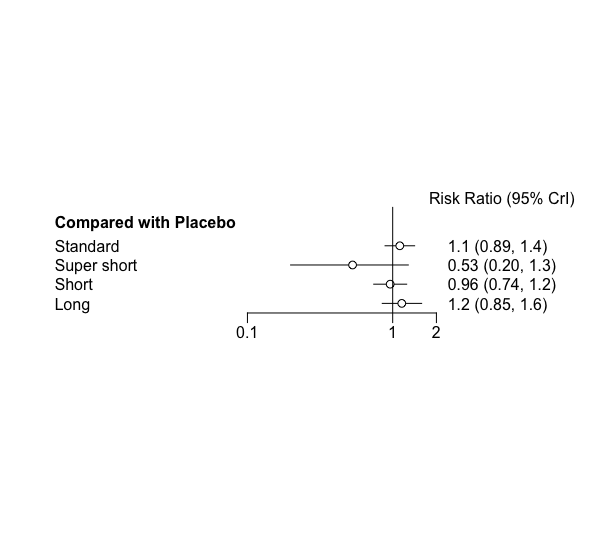


**Figure SIV-3.1 Relative Effect Forest Plot of Adverse events(omitted the mixed ones) (baseline: placebo)**

**Abbreviation:** CrI, credible interval.

- 1. **League table**

**Table SIV-3.2 League Table of Adverse events(omitted the mixed ones)**

| Standard |  |  |  |  |
| --- | --- | --- | --- | --- |
| 2.12 (0.9, 5.52) | Super short |  |  |  |
| **1.16 (1.02, 1.34)** | 0.55 (0.21, 1.29) | Short |  |  |
| 0.97 (0.76, 1.24) | 0.46 (0.17, 1.09) | 0.83 (0.67, 1.03) | Long |  |
| 1.12 (0.89, 1.42) | 0.53 (0.2, 1.28) | 0.96 (0.74, 1.25) | 1.16 (0.85, 1.58) | Placebo |

- 1. **Rank of probability**


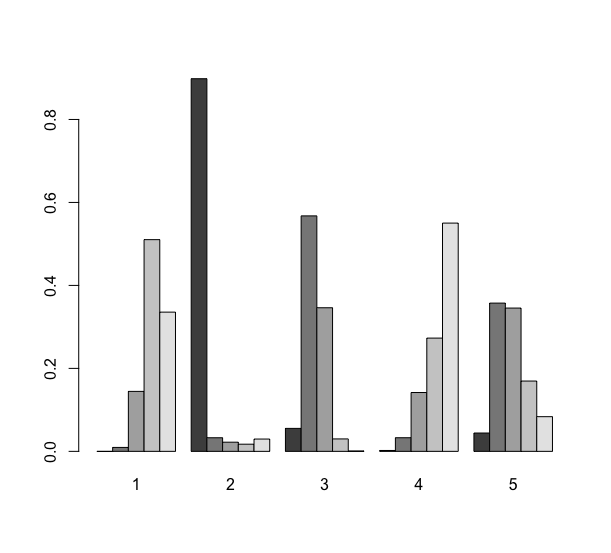


**Figure SIV-3.3 Bar chart of rank of probability of adverse events(omitted the mixed ones).**

**Notes:** 1, standard treating course; 2, super short treating course; 3, short course; 4, long course;5, placebo.

- 1. **SUCRA rank of probability**

**Table SIV-3.4 SUCRA rank of probability of adverse events(omitted the mixed ones).**

| Intervention | SUCRA |
| --- | --- |
| Standard | 10.40% |
| Super short | 95.37% |
| Short | 59.55% |
| Long | 35.34% |
| Placebo | 49.33% |

**Abbreviation:** SUCRA, surface area under the cumulative ranking curve.

1. **Quality Control**
   1. **Convergence test: PSRF value**


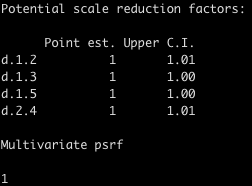


**Figure SIV-4.1 PSRF of the Bayesian analysis of adverse events(omitted the mixed ones)**

**Abbreviation:** PSRF, Potential scale reduction factors.

**Note:** The closer the PSRF value is to 1, the better. >1.05 indicates unsatisfactory convergence.

- 1. **Consistency hypothesis: inconsistency test.**
     1. **Function used:** *mtc.nodesplit*
     2. **Results:**

**
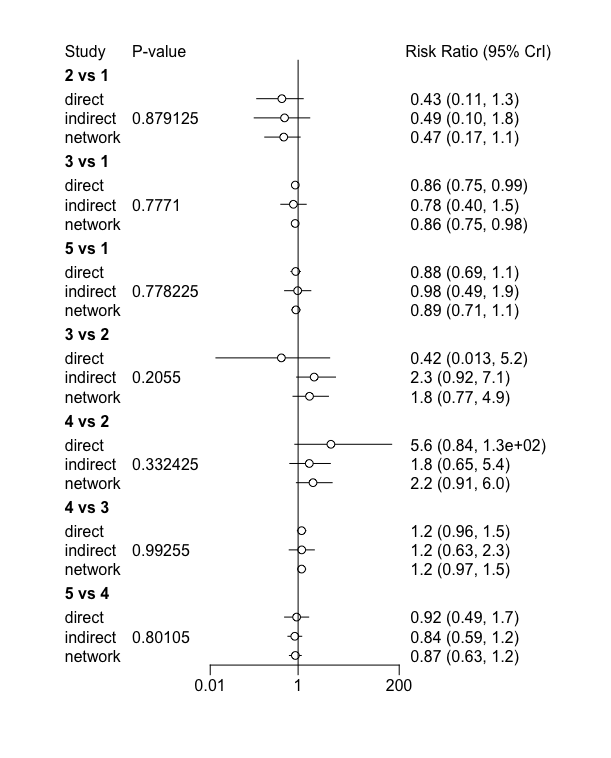
**

**Figure SIV-4.2.2 Inconsistency test of the Bayesian analysis of adverse events(omitted the mixed ones)**

**Note:** 1, standard treating course; 2, super short treating course; 3, short course; 4, long course;5, placebo. *P* value > 0.05 indicates that there is no significant inconsistency which means the direct, indirect and network comparison is consistent.

- 1. **Homogeneity hypothesis: Heterogeneity analysis**
     1. **Function used:** *mtc.anohe*
     2. **Results**


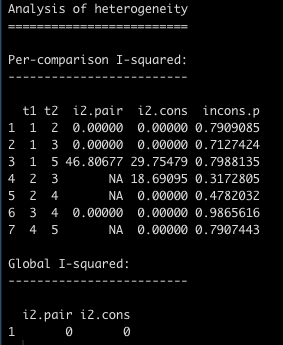


**Figure SIV-4.3.2 Heterogeneity analysis of the Bayesian analysis of adverse events(omitted the mixed ones)**

**Note:** From the figure we could see, there exists no significant heterogeneity between any two arms, which allows the use of fixed effect model to estimate the pooling results.
